# Does the natural product, honokiol, have value in the battle against osimertinib resistance?

**DOI:** 10.18632/oncoscience.517

**Published:** 2020-09-09

**Authors:** Karin A. Vallega, Shi-Yong Sun

**Affiliations:** ^1^Department of Hematology and Medical Oncology, Emory University School of Medicine and Winship Cancer Institute, Atlanta, GA, USA

**Keywords:** natural products, honokiol, osimertinib, resistance, EGFR mutation, lung cancer

Worldwide, lung cancer is the leading cause of cancer deaths. The invention of epidermal growth factor receptor-tyrosine kinase inhibitors (EGFR-TKIs) have revolutionized treatment for non-small cell lung cancer (NSCLC) patients with activating EGFR mutations. EGFR-TKIs are the first targeted therapy available for lung cancer, and show improvement over chemotherapy as a first-line treatment [1]. Unfortunately, the emergence of acquired resistance limits long-term benefits for patients. Resistance to early generations of EGFR-TKIs are predominately due to the appearance of the T790M resistance mutation, which accounts for 41-62% of relapse cases [1]. Third-generation EGFR-TKIs, such as osimertinib (AZD9291/TAGRISSO^TM^), were developed to target the acquired T790M mutation and the common activated mutations, such as 19del and L858R, while limiting activity against wild-type EGFR. Osimertinib is now approved as a second line treatment after acquired resistance to earlier EGFR-TKIs due to the T790 mutation, and as a first line treatment for NSCLC patients with the common activated EGFR mutations. It shows improved progression-free survival compared to earlier generation EGFR-TKIs [2] with the advantage of significantly improving overall survival [3]. However, acquired resistance to osimertinib has also emerged. On average, patients relapsed after 18.9 months when using osimertinib as a first-line treatment, and 10.1 months when used as a second-line treatment [2, 4]. Therefore, it is imperative to develop novel strategies to overcome osimertinib acquired resistance and increase patient survival and quality of life.

Many current cancer treatments are derived from or made to mimic natural products that have been used as traditional medicine for centuries [5]. Honokiol (HNK) is a compound found in the leaves and bark of Magnolia trees [5]. HNK has been used in traditional Chinese and Japanese medicine for a variety of issues, including gastrointestinal disorders, strokes, allergies, anxiety and depression [5]. HNK has a range of biological activity, including anti-oxidative, anti-inflammatory, and anti-microbial effects [5]. Preclinical studies have demonstrated that HNK has antitumor effects on several human cancers, including lung, breast, prostate, colon, skin, and gall bladder [5].

Our recent study has shown that HNK and its derivatives possess promising activity in overcoming osimertinib acquired resistance in a preclinical setting [6]. In this study, the combination of HNK and osimertinib synergistically decreased survival and colony formation in osimertinib-resistant cell lines with different resistance mechanisms. The combination also significantly enhanced induction of apoptosis compared to each agent alone. Therefore, it is apparent that HNK re-sensitizes osimertinib-resistant cell lines to osimertinib. The combination facilitated degradation of Mcl-1, resulting in effective reduction of Mcl-1 levels in different osimertinib-resistant cell lines. Enforced expression of ectopic Mcl-1 in an osimertinib-resistant cell line compromised the ability of osimertinib and HNK combination to enhance induction of apoptosis. It appears that Mcl-1 reduction is an important mechanism accounting for enhanced induction of apoptosis by the combination in osimertinib-resistant cells. Bim elevation may also play a role in mediating induction of apoptosis by the combination in some resistant cell lines, since Bim levels were increased in one of the tested cell lines by the combination. Additionally, Bim knockout in this cell line significantly reduced cell response to the combination. Beyond these *in vitro* findings, the combination of HNK and osimertinib also effectively inhibited the growth of osimertinib-resistant tumors *in vivo*, causing smaller tumor size and weight compared to vehicle controls or single agent treatment. The combination did not decrease the body weight of mice, indicating that this combination is well-tolerated by the mice. 

As a natural product, HNK has been used as a nutritional supplement in humans; therefore, it is well-tolerated and safe [7]. Acquired C797S mutation is now a defined mechanism for the emergence of acquired resistance to osimertinib and accounts for 20-30% of resistant cases when osimertinib is used as a second-line treatment [8]. There are no effective options for the treatment of resistant tumors with triple mutations of EGFR at 19del, T790M and C797S. The intriguing finding in our study is that the HNK and osimertinib combination is effective against the growth of osimertinib-resistant tumors harboring EGFR 19del, T790M and C797S triple mutations. Approximately 33% of patients with EGFR-mutant NSCLC develop brain metastasis [9], which is the main cause of mortality in this population. While osimertinib has improved efficacy against brain metastases [10], another valuable feature of HNK is that it can readily cross the blood-brain barrier and the blood-cerebrospinal fluid barrier [11], making it a suitable candidate for combining with osimertinib. Hence, HNK has unique features to meet the urgent clinical need of overcoming osimertinib resistance.

One potential drawback or concern regarding the use of natural products is their relatively weak biological activities. In our study, we have shown that CAz-p, a derivative of HNK, exerted similar effects as HNK in decreasing the survival and enhancing the induction of apoptosis of several osimertinib-resistant cell lines, including one with EGFR triple mutations, at a much lower concentration range than HNK. Therefore, it is possible to use HNK and CAz-p as lead compounds for developing novel agents with optimized efficacy and pharmacological characteristics to overcome osimertinib acquired resistance.

Our findings have identified HNK and its derivatives as potential candidates for overcoming acquired resistance to osimertinib caused by varied mechanisms such as C797S mutation, providing strong preclinical support for testing this potential strategy for overcoming acquired osimertinib resistance in the clinic. Further studies to fully understand the underlying molecular mechanisms and to develop HNK derivatives that can overcome osimertinib acquired resistance are thus warranted.

**Figure 1:**
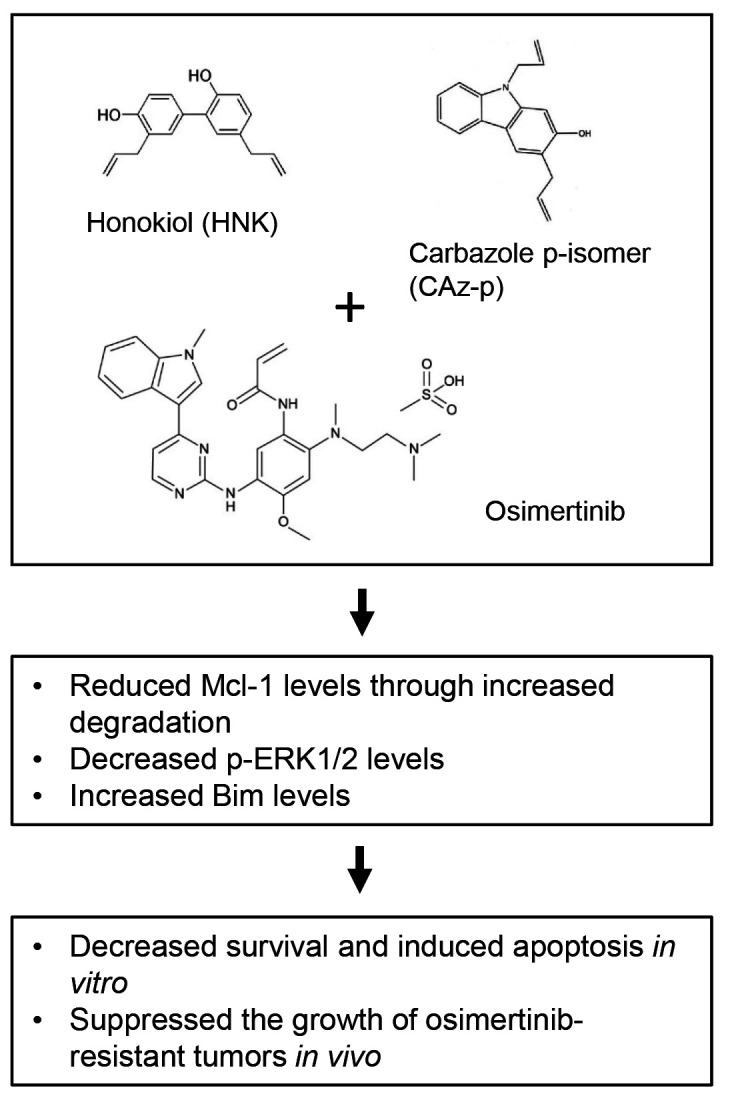
Schematic summary of HNK and osimertinib combination against osimertinib resistance.

Cancer treatment failure mostly involves the insensitivity of a small, heterogeneous fraction of cancer cells within the tumor mass, endowed with extraordinary plasticity and the ability to self-renew and metastasize [1, 2]. These cells, termed cancer stem cells (CSCs) are, in general, resistant to conventional anti-cancer treatments [1, 2]. (Figure 1) resumes current approaches for CSC disarming.
